# Complete Genome Sequence of Murine Pneumotropic Virus (*Polyomaviridae*) Clone pKV(37-1)

**DOI:** 10.1128/genomeA.00404-16

**Published:** 2016-05-19

**Authors:** Jane E. Libbey, Robert S. Fujinami

**Affiliations:** Department of Pathology, University of Utah School of Medicine, Salt Lake City, Utah, USA

## Abstract

The murine pneumotropic virus genome encoded by the pKV(37-1) clone was sequenced to completion. The regulatory region harbored a mutation not previously reported. The protein coding regions (large and small T antigens, viral proteins 1 to 3) showed multiple regions of high amino acid identity to the human, simian, and bovine polyomaviruses.

## GENOME ANNOUNCEMENT

Murine pneumotropic virus (MPtV), previously Kilham polyomavirus, was first isolated/described over 50 years ago (1, 2). The natural host of MPtV is *Mus musculus* (1). Acute severe interstitial pneumonia is seen in newborn mice, while a clinically inapparent infection is followed by lifelong persistence in immunocompetent mice (3, 4). The MPtV complete genome was originally sequenced (GenBank accession number NC_001505) to elucidate the genomic organization and relationship to *Polyomaviridae* (5) and more recently sequenced (KT987216 to KT987218) to explore the diversity of MPtV in wild mice (6).

We sequenced the plasmid, pKV(37-1), encoding the entire MPtV genome inserted into the PstI site of pBR322, obtained from the American Type Culture Collection (ATCC) (7), as the plasmid sequence was not previously reported. The plasmid was propagated in and isolated from *Escherichia coli* using standard molecular biology techniques (8) and was directly sequenced without prior PCR amplification. All sequencing was done by the Sequencing Facility at the University of Utah.

Here we report the presence of a novel mutation in the plasmid. We found the noncoding regulatory region, which contains the origin of viral DNA replication as well as all transcriptional regulatory sequences, to be most consistent with one of the variants ([9]; AJ517509), which contained a 220-bp insertion at nucleotide 142 (numbering according to reference 9) of a cellular transposable element (type A,*in*A) in combination with a small deletion (*dl*143-148). However, the plasmid regulatory region also contained the G272T transversion ([9]; AJ517508), and surprisingly, a small deletion (*dl*91-96) not previously characterized. Although the regulatory region of MPtV has been shown to have many variants, the plasmid distributed by ATCC may not be an appropriate representative of these variants due to this additional deletion (*dl*91-96). Extensive characterization of the regulatory region in virus DNA preparations failed to demonstrate the presence of this particular deletion in any variants (9).

The protein coding region showed multiple regions which generally had higher amino acid (aa) identity to the human (JC and BK), simian (SV40), and bovine polyomaviruses than to the MPtV sequence (NC_001505) or to murine polyomavirus (MPyV). For large T antigen, aa 126 to 135 had 33.3% identity with both MPyV and MPtV (NC_001505), aa 193 to 212 had 44.4% identity with JC and BK, and aa 341 to 365 and 461 to 495 had 64.0% and 76.2%, respectively, identity with SV40. Viral protein (VP)1 aa 9 to 24 had 38.1% identity with bovine polyomavirus, aa 162 to 170 had 55.6% identity with JC, BK, and SV40, aa 278 to 294 had 88.9% identity with bovine polyomavirus, and aa 306 to 318 had 57.9% identity with MPyV. VP2 and VP3 aa 212 to 238 had 48.4% identity with bovine polyomavirus. The carboxyterminal ends of the large T antigen and VP1-3 were highly variable across all of the polyomaviruses. Only the small T antigen had high sequence identity with MPtV (NC_001505).

The MPtV genomes from wild mice (KT987216 to KT987218) had only a 1% nucleotide difference compared to the MPtV genome encoded on pKV(37-1), as determined by us (6).

### Nucleotide sequence accession number. 

The MPtV insert of pKV(37-1) has been deposited in GenBank under accession number EF186666.
